# Computation of Fresnel Integrals. II

**DOI:** 10.6028/jres.105.049

**Published:** 2000-08-01

**Authors:** Klaus D. Mielenz

**Affiliations:** Oakland, MD 21550

**Keywords:** computation, Fresnel integrals, rational approximations, series expansions

## Abstract

This paper describes an improved method for computing Fresnel integrals with an error of less than 1 × 10^−9^. The method is based on a known approximate formula for a different integral which is due to Boersma and referenced by Abramowitz and Stegun.

## 1. Improved Computation of Fresnel Integrals

In a previous paper [1], this author presented formulas for numerical computations of the Fresnel cosine and sine integrals,

$$\begin{array}{l} C(x)=\int_{0}^{x}dt\cos\left(\frac{\pi }{2}t^{2}\right)\\ \;=\frac{1}{2}+f(x)\sin\left(\frac{\pi }{2}x^{2}\right)-g(x)\cos\left(\frac{\pi }{2}x^{2}\right), \end{array}$$ (1a)

$$\begin{array}{l} S(x)=\int_{0}^{x}dt\sin\left(\frac{\pi }{2}t^{2}\right)\\ \;=\frac{1}{2}-f(x)\cos\left(\frac{\pi }{2}x^{2}\right)-g(x)\sin\left(\frac{\pi }{2}x^{2}\right), \end{array}$$ (1b)

to six significant figures by using the first three terms of the Taylor expansions of *C*(*x*) and *S*(*x*) for |*x*| ≤ 0.6, the first three terms of the asymptotic expansions of the auxiliary functions *f*(*x*) and *g*(*x*) for |*x*| ≥3, and modified rational approximations of *f*(*x*) and *g*(*x*) for the mid range. These formulas proved hard to use because they involve too many numerical constants.

It was found subsequently that a simpler and more accurate method of computation can be based on a formula derived by Boersma [2],

$$\begin{array}{c} \int_{0}^{u}\frac{d\tau e^{-i\tau }}{\sqrt{2\pi \tau }}=\frac{1-i}{2}+e^{-iu}\sqrt{\frac{4}{u}}\sum_{n=0}^{11}(p_{n}+iq_{n}){\left(\frac{4}{u}\right)}^{n},\\ u\geq 4, \end{array}$$ (2a)

where *p_n_* and *q_n_* are numerical constants tabulated in Ref. [2] and the notation has been changed in order to avoid confusion with symbols used elsewhere in this paper. On substituting *t* = πτ^2^/2 and *x* = π*u*^2^/2 this is transformed into

$$\begin{array}{c} \int_{0}^{x}dte^{-i\pi t^{2}/2}=C(x)-iS(x)=\frac{1-i}{2}\\ -e^{-i\pi x^{2}/2}\sum_{n=0}^{11}\frac{g_{n}-if_{n}}{\chi^{2n+1}},\;x\geq 1.6, \end{array}$$ (2b)

$$f_{n}={\left(8/\pi \right)}^{n+1/2}q_{n},\;g_{n}=-{\left(8/\pi \right)}^{n+1/2}p_{n}.$$ (2c)

Hence it follows by separation of real and imaginary parts and comparison with Eqs. (1a) and (1b) that

$$f(x)=\sum_{n=0}^{11}f_{n}\;\chi^{-2n-1},\;g(x)=\sum_{n=0}^{11}g_{n}\;\chi^{-2n-1}.$$ (2d)

The required coefficients, as computed from Boersma’s data and Eq. (2c), are listed in Table 1. Equation (2d) was tested by computing sample values of *C*(*x*) and *S*(*x*) and comparing them to the values tabulated in Ref. [3] to eight digits. The agreement was perfect, which is consistent with Boersma’s statement that the error of Eq. (2a) is less than 5×10^−10^.

The above method is implicitly contained in Ref. [3], which mentioned Boersma’s paper as well as the relationship between the integrals in Eqs. (1a), (1b), and (2a). Boersma also gave an approximation formula similar to Eq. (2a) for |*u*| ≤ 4, or |*x*| ≤ 1.6. This was not used in this work because in this range it is simpler to compute *C*(*x*) and *S*(*x*) by using their Taylor expansions [3],

$$\begin{array}{c} C(x)=\sum_{n=0}^{\infty }c_{n}\;\chi^{4n+1},\;c_{0}=1,\\ c_{n+1}=\frac{-\pi^{2}(4n+1)c_{n}}{4(2n+1)(2n+2)(4n+5)}, \end{array}$$ (3a)

$$\begin{array}{c} S(x)=\sum_{n=0}^{\infty }s_{n}\;\chi^{4n+3},\;s_{0}=\frac{\pi }{6},\\ s_{n+1}=\frac{-\pi^{2}(4n+1)s_{n}}{4(2n+2)(2n+3)(4n+7)}. \end{array}$$ (3b)

These give results with errors less than 6×10^−10^ for |*x*|≤1.6 if the first 11 terms of the expansions are carried.

Software and algorithms for computing Fresnel integrals in Fortran and C (not based on this paper) are also available on the Internet [4,5].

## Figures and Tables

**Table 1:** Numerical values of *f_n_* and *g_n_*

*n*	*f_n_*	*g_n_*
0	0.318309844	0
1	9.34626E-08	0.101321519
2	−0.09676631	−4.07292E-05
3	0.000606222	−0.152068115
4	0.325539361	−0.046292605
5	0.325206461	1.622793598
6	−7.450551455	−5.199186089
7	32.20380908	7.477942354
8	−78.8035274	−0.695291507
9	118.5343352	−15.10996796
10	−102.4339798	22.28401942
11	39.06207702	−10.89968491
